# Impact of wall shear stress on initial bacterial adhesion in rotating annular reactor

**DOI:** 10.1371/journal.pone.0172113

**Published:** 2017-02-16

**Authors:** Thibaut Saur, Emilie Morin, Frédéric Habouzit, Nicolas Bernet, Renaud Escudié

**Affiliations:** LBE, INRA, Narbonne, France; University of Porto, PORTUGAL

## Abstract

The objective of this study was to investigate the bacterial adhesion under different wall shear stresses in turbulent flow and using a diverse bacterial consortium. A better understanding of the mechanisms governing microbial adhesion can be useful in diverse domains such as industrial processes, medical fields or environmental biotechnologies. The impact of wall shear stress—four values ranging from 0.09 to 7.3 Pa on polypropylene (PP) and polyvinyl chloride (PVC)—was carried out in rotating annular reactors to evaluate the adhesion in terms of morphological and microbiological structures. A diverse inoculum consisting of activated sludge was used. Epifluorescence microscopy was used to quantitatively and qualitatively characterize the adhesion. Attached bacterial communities were assessed by molecular fingerprinting profiles (CE-SSCP). It has been demonstrated that wall shear stress had a strong impact on both quantitative and qualitative aspects of the bacterial adhesion. ANOVA tests also demonstrated the significant impact of wall shear stress on all three tested morphological parameters (surface coverage, number of objects and size of objects) (p-values < 2.10^−16^). High wall shear stresses increased the quantity of attached bacteria but also altered their spatial distribution on the substratum surface. As the shear increased, aggregates or clusters appeared and their size grew when increasing the shears. Concerning the microbiological composition, the adhered bacterial communities changed gradually with the applied shear.

## 1. Introduction

Microbial adhesion, as the first step of biofilm formation, constitutes a critical stage in biofilm development and management [1]. This process is of great interest in a wide range of domains such as environmental biotechnology [2–4], different medical fields [5,6] or industrial processes [7–10]. A better understanding of the impact of wall shear stress on the microbial adhesion is crucial to prevent sanitary and economic issues associated to the development of detrimental biofilms. In addition, it could help to develop and to optimize beneficial biofilm applications, such as water and wastewater treatment, bioremediation or industrial biotechnology.

Hydrodynamic forces play a key role in microbial adhesion [1,11]. Adhesion involves the transport of bacteria towards the substratum surface and their attachment on it. The hydrodynamic conditions can have an ambivalent effect on adhesion mechanisms. On one hand, a low wall shear stress applied on the biofilm substratum may limit the detachment forces and may promote bacterial adhesion. On the other hand, increasing the mixing efficiency and the convective transport can also promote microbial adhesion, as it facilitates the access of bacteria to the substratum. However, in the turbulent flows applied in industrial water systems, a higher mixing efficiency is associated with higher wall shear stresses, preventing a long-lasting bacterial adhesion. It was indeed reported not only a decreasing number of adhesion events while increasing the shear, but also an increasing number of firmly attached bacteria [12]. Also, another study obtained the highest level of adhesion in microfluidic channel for intermediate values of shear [13]. Thus, despite the large number of studies dealing with the impact of shear stress on adhesion coverage, many aspects remain unclear.

In addition, shear can also qualitatively impact the attached bacterial communities. To our knowledge, only few studies have investigated the adhesion step of real and diverse microbial communities in turbulent flows [4,14]. It could be interesting to investigate whether or not bacterial interactions can influence the adhesion step and the impact of the hydrodynamic conditions on it. Also, to know whether or not shear can be used to control or select the bacterial communities during the adhesion step is of great interest. Such control could help to avoid pathogens in industrial and medical biofilms or to promote the settlement of specific strategic populations in environmental bioprocesses (e.g., methanogenic *Archaea*, Anammox bacteria…).

Other than the wall shear stress, microbial adhesion has also been widely reported to be affected by the substratum characteristics. This second parameter can influence the balance between substratum-micro-organisms attachment forces and the detachment forces exerted by the hydrodynamic stress. Indeed, all microbial species have their own intrinsic features that determine their attachment ability. This ability is related to the substratum properties, such as surface energy, hydrophobicity or roughness. In addition, the affinity of a microorganism to a given substratum material plays a key role in both quantitative and qualitative properties of adhesion. For example, different types of substrata in terms of surface energy and hydrophobicity were characterized and significantly different adhesion of *Listeria monocytogenes* were obtained depending on the substratum [7]. In addition, significant differences in terms of bacterial surface coverage and structure of bacterial communities attached were found when seven materials (including glass, plastics and stainless steel) were compared [4].

In the literature, most of the research has been devoted to the impact of shear on biofilm adhesion in pure culture and under laminar flow and/or low wall shear stresses [12,15,16]. Nevertheless, in industrial and man-made processes as well as in natural environments, adhesion is mediated by many different strains of microorganisms [5,7]. Moreover, the complex hydraulic circuits found in industrial processes make the range of encountered shears and Reynolds numbers very wide [10,17]. Thus, the novelty of this work lies on the application of working conditions more relevant to mimic industrial process, in terms of hydrodynamic conditions and bacterial community. To fill this research gap, a wide range of wall shear stresses, 0.09–7.3 Pa, equivalent to a range of wall shear rates of 86–7300 s^-1^, were tested in this study. This range reflects the wide variety of laboratory and industrial hydrodynamic systems in which biofilms can be found [17,18]. A diverse inoculum consisting of activated sludge collected in a wastewater treatment plant was used. To evaluate the impacts of the shear stress, the microbial communities and the adhesion quantification and distribution were analyzed on two different materials as substratum. Polypropylene (PP) and polyvinyl chloride (PVC) were chosen as materials, as they have been found to have different adhesion characteristics [4]. Moreover, both materials are relevant in industrial and environmental bioprocesses (tubing, biofilm carriers…). Thus, the objective of this work was to assess the impact of the wall shear stress on the adhesion process of a complex and diverse inoculum under turbulent hydrodynamic conditions, parameters being more relevant to mimic bioprocess engineering issues.

## 2. Methods

### 2.1 Reactor characteristics

Rotating annular reactors–also named Couette-Taylor reactors (CTR)–from Biosurface Technologies Cord, Bozeman, USA (model 1320LJ) were used to investigate the microbial adhesion. These reactors present several advantages. The liquid phase is completely mixed, ensuring an uniform distribution of bacteria in the bulk phase [19]. The constant distribution of the wall shear stress makes also this reactor very suitable for the purposes of this work. Furthermore, hydrodynamic conditions and flow regimes are well-defined [20,21] and shear stress can be accurately estimated [14,22]. Also, high Reynolds numbers ensure the establishment of a turbulent flow.

Reactors consisted of two concentric glass cylinders, a rotating inner cylinder and a non-rotating outer cylinder. The inner cylinder was driven by a motor whose rotational speed could be selected–from 25 to 350 rpm–allowing the selection of different wall shear stresses. The radius of the inner cylinder (r_i_) and the outer cylinder (r_e_) were 70 and 98 mm respectively. The gap (δ) between the two cylinders was 28 mm for a 3 liters volume. Up to 20 removable slides could be placed on the inner cylinder and they were beveled to perfectly fit slots present on the inner cylinder thus minimizing local secondary flow. The available surface area for adhesion was 18.75 cm^2^ (150 mm x 12.5 mm) per slide.

Slides of polyvinyl chloride (PVC) and polypropylene (PP) were used in the present study. They were previously characterized in terms of surface energies and roughness [4]. Table 1 presents the surface energies for the PP and PVC slides. The roughness characteristics are not presented because very similar results were obtained for both PP and PVC (average roughnesses being 0.041 and 0.038 μm, respectively). Detailed data are available in the literature [4]. Concerning the characterization of the surface energy, the contact angles measurement method was used [23]. A goniometer (G10, Krüss) measured the contact angles on cleaned slides with deionized water, formamide and diiodomethane.

**Table 1:** Characterization of the two materials used as substrata: polyvinyl chloride (PVC) and polypropylene (PP).

	Surface energies (mJ.m^-^^2^)		
	γ	γ^LW^	γ^AB^
PP	35.6±1.7	34.1±1.4	1.6±1.0
PVC	51.2±1.9	43.1±1.5	8.1±1.2

γ surface energy; γLW, Lifshitz–Van der Waals component; γAB, polar component. Data extracted from [4].

### 2.2. Hydrodynamics and wall shear stress

Hydrodynamic conditions and flow regimes in CTR are well documented. The characteristic of the flow regimes in a CTR have been well described elsewhere [20,21,24] and are defined by the dimensionless number of Taylor (Ta):

$$Ta=\frac{\Omega \;r_{i}^{\frac{1}{2}}\;\delta^{\frac{3}{2}}}{\nu }$$ (1)

where r_i_ is the radius of the inner cylinder (m) δ is the gap between the two cylinders (m), Ω is the rotational speed of the inner cylinder (rad.s^-1^) and ν is the kinematic viscosity of water (m^2^.s^-1^). Both numbers are often described as the ratio between inertial forces–centrifugal forces in CTR–and viscous forces. They can also be used to determine the turbulence onset.

Calculations based on literature [20,21,24,25] highlighted a Ta = 500 for the onset of a turbulent flow. Even at the lowest rotational speed of 25 rpm on our system, Ta equaled 3.245, ensuring that a turbulent flow was effective during the microbial adhesion tests. The wall shear stress (τ) was also well defined in the CTR. As described and applied in the literature [14,22], the following equations can be used to estimate τ:

$$\tau =2.13\frac{{\left(\frac{r_{i}}{r_{e}}\right)}^{\frac{3}{2}}}{{\left(1-\frac{r_{i}}{r_{e}}\right)}^{\frac{7}{4}}}\;{Re}^{1.445}\frac{\rho \nu^{2}}{2\pi r_{i}^{2}}\;\text{for}\;\text{Re}>800$$ (2a)

$$\tau =0.113\frac{{\left(\frac{r_{i}}{r_{e}}\right)}^{\frac{3}{2}}}{{\left(1-\frac{r_{i}}{r_{e}}\right)}^{\frac{7}{4}}}\;{Re}^{1.764}\frac{\rho \nu^{2}}{2\pi r_{i}^{2}}\;\text{for}\;\text{Re}>10^{4}$$ (2b)

where ρ is the density of water (kg.m^-3^), r_e_ the radius of the outer cylinder (m) and Re the dimensionless number of Reynolds defined as:

$$Re=\frac{\Omega r_{i}\delta }{\nu }$$ (3)

For the lowest available speed of 25 rpm, Re equaled 5,131 and the wall shear stress on the inner cylinder was 0.09 Pa. For the highest rotational speed of 350 rpm, τ equaled 7.3 Pa. Two intermediate wall shear stresses were also tested: 3.7 Pa, which corresponds to the center on a linear scale between the highest and the lowest shears and 0.79 Pa, which corresponds to the center on a logarithmic scale between the highest and the lowest shears. These four wall shear stresses are equivalent to 86–787–3700 and 7300 s^-1^ wall shear rates (5,000 < Re < 72,000).

### 2.3. Microbial adhesion protocol

The inoculum of the reactors was the supernatant of an activated sludge process. For each experiment (Var 1 and 2, Shear 1 and 2 and Mat experiments), twenty liters of fresh aerobic activated sludge were collected the day before from a wastewater treatment plant (Armissan, France). The activated sludge was settled for one hour and ten liters of the supernatant was recovered in a glass bottle. This procedure was necessary in order to remove the flocs of sludge from the inoculum. The deposition of these flocs on the slides strongly compromised the microscopic observation after the DAPI treatment. The supernatant was then fed for 24h in aerobic conditions with a medium made of yeast extract, meat extract and peptone in proportions 1/1/1. The amount of substrate to be added was calculated to reach an initial Chemical Oxygen Demand (COD) over Volatile Suspended Solids (VSS) ratio (S/X) of 0.1g_COD_.g_VSS_^-1^, corresponding to a low organic load. By this procedure, bacteria were able to grow, obtaining an active and concentrated inoculum. Before starting the experiments, it was checked that a low soluble COD concentration had been reached in the inoculum. Values between 30 and 80 mg.L^-1^ were obtained. These low values and the short contact time during the experiment allowed to prevent a significant bacterial growth during the adhesion process.

Before each experiment, the reactors and the new slides were sterilized with a detergent solution (RBS 35, Traitements Chimiques de Surfaces, Frelinghien, France) used at a concentration of 5% vol/vol. They were then rinsed with autoclaved water. First, the rotation of the inner cylinder was turned on at the determined speed and then 3 liters of inoculum were pumped into the reactor. The preparation steps were carried out in that precise order to avoid any contact between the inoculum and the slides when no shear was applied. Similarly, at the end of the one-hour contact time between the inoculum and the slides, the reactors were systematically washed out to remove the planktonic bacteria before the rotation of the cylinder was switched off. The slides extraction from the reactor was a time consuming step as each slide was treated right after its extraction for either morphological or microbiological characterizations. To avoid any adhesion during this timeframe while no shear was applied on the slides, this wash-out protocol was required. It ensured that the observed adhesion necessarily occurred when shear was applied on the slides. Thus, after a one-hour contact time between bacteria and slides, the reactors were washed out for one hour with a sterile solution continuously pumped in the reactors. This solution consisted of the outlet of the wastewater treatment plant sampled the day before the experiment as well. Before its addition in the reactor, this solution was filtered at 30 μm on a nylon filtration cloth (Fisherbrand, Fisher Scientific) to avoid abrasion on slides and it was autoclaved to ensure that it was sterile. Eight volumes of the solution were added in the reactors corresponding to a flow rate of 400 ml.h^-1^. By this way, almost no bacteria remained in the liquid phase when rotation was stopped (> 3 log decrease in planktonic bacteria concentration). Growth might be possible during the wash-out step but should be really limited given the COD concentration and composition (concentration lower than 40 mg/L and including recalcitrant COD) at the outlet of a low-load activated sludge process as the one collected. Slides were finally removed from the reactor and rinsed by immersion in a 0.2 μm filtered outlet of the wastewater treatment plant before being processed either for morphological or microbiological characterizations.

### 2.4. Morphological structure analysis

#### 2.4.1. Image acquisition

All the solutions used for staining the slides and for image acquisition were prepared with a 0.2 μm filtered outlet of the wastewater treatment plant. This allowed to minimize changes in osmotic and ionic forces and to avoid the detachment of bacteria on the substratum.

Once carefully removed from the reactor, the slides were rinsed and stained in a DAPI (4’,6-diamidino-2-phenylindole) solution. Microscopic observations were done with an Olympus BX 60-F microscope. The acquisition was realized with a CCD Nikon Digital Camera DXM 1200F. Images with a dimension of 1280x1024 pixels^2^, equivalent to 0.013 mm^2^, were acquired in lossless RGB tif format. The acquisition parameters such as exposure time, sensitivity, use of UV filter (excitation filter: 330–385 nm, barrier filter: 420 nm) were kept constant for a given material and a given experiment. Sixty images equivalent to 0.78 mm^2^ were recorded per slide.

#### 2.4.2. Image processing

Images were processed using the software Image J [26]. First of all, the function “Subtract Background” was used to remove heterogeneous light distribution. Images were then binarized using the “Color threshold” and “Binary” functions. Finally, the functions “Close” and “Open” were applied in order to fill small holes and remove isolated pixels considered as noise.

From these black and white images, three parameters were calculated:

- surface coverage defined here as the fraction of black pixels over the total pixels (black and white pixels),
- number of particles, whatever their size and shape,
- average size of particles, whatever their shape.

Statistical analyses were then performed on these parameters with the statistical software R 2.15. Boxplots were used to control image processing quality and remove misprocessed pictures. Indeed, the image processing might be prone to mistakes. Also, some DAPI crystals or aggregates may have been recorded by mistake. Boxplots were then used as a way to control pictures quality and to check whether or not a problem occurred. If a problem was identified, then the picture was visually checked and eliminated from the dataset. ANOVA and t-tests were performed to assess the statistical significance of our results (tests were considered significant for p-values < 5x10^-2^).

### 2.5. Microbiological structure analysis

#### 2.5.1. DNA extraction

The slides were rinsed by immersion in a 0.2 μm filtered outlet of the wastewater treatment plant and scraped with moistened sterile quartz wool. This bacteria harvesting method has been tested by staining scraped slides–same protocol as the one described in the image acquisition section–to check that the adhered bacteria were removed from the substratum. The wool was frozen at -20°C until DNA extraction was performed using the QIAamp DNA Stool Minit Kit (Qiagen, Germantown, MD, USA), in accordance with manufacturer instructions. The quantity and purity of DNA was checked by spectrometry. In average, 21 ng.μL^-1^ were recovered from the quartz wool samples.

#### 2.5.2. PCR-SSCP

Bacterial communities were characterized by PCR-SSCP (Single Strand Conformation Polymorphism) fingerprinting technique [14,27]. The V3 region of the 16S rRNA gene were amplified for *Bacteria* with a fluorescent dye-labelled W49F primer (AGGTCCAGACTCCTACGGG) and W104R primer (TTACCGCGGCTGCTGGCAC) [14,27]. During the amplication process, the amplified DNA also inherits the fluorochrome from the dyed-labelled primer allowing its detection by fluorescence. The amplification thermal cycle was as follows: an initial 2 min denaturing step at 94°C, 25 cycles at 94°C for 30 s, at 61°C for 30 s and at 72°C for 30 s and a final 10 min elongation step at 72°C. Approximately 0.15 ng of PCR products were then used for the capillary electropheresis single-strand conformation polymorphism (CE-SSCP), as described by Rochex *et al*. (2008) [14]. This method allows to obtain a single strand conformation of DNA which depends on the sequence. Briefly, 1 μL of PCR products (diluted if required) was placed in a mixture of formamide (18.925 μL) and a size standard (0.075μL) (ROX GeneScan 400, Applied Biosystems). The mix was denaturated at 95°C for 5 min and rapidly cooled in ice. Single stranded DNA were then separated by capillary electrophoresis by using ABI 3130 genetic analyzer (Applied Biosystems).

#### 2.5.3. Fingerprinting profile statistical analysis

As described by Milferstedt *et al*. *(2013)* [28], bacterial fingerprints from the CE-SSCP profiles were processed with the Statfingerprints package [29] implemented in the software R 2.12 [30]. They were aligned based on the ROX internal size standards and normalized. To compare SSCP profiles more easily, Principal Component Analysis (PCA) was performed by using the package vegan designed for community ecology studies [31]. The PCA allowed to aggregate the differences between all SSCP profiles, i.e. their genetic differences, and to summarize most of the information in a one or two dimensional space. This procedure sums up the information contained in the SSCP profiles and makes the representation of several samples on the same graph.

### 2.6. Strategy for microbial adhesion study

The effect of wall shear stress on microbial adhesion was studied in three successive phases (Table 2). For the experiments with more than one shear tested, up to three CTR were used simultaneously, each exhibiting a given shear stress. Both morphological–spatial pattern of adhesion–and microbiological–bacterial communities–structures were characterized. These two characterizations being time-consuming, a maximum of twenty slides per experiment were processed. As a consequence, the number of slides per condition had to be adapted depending on the number of running CTR and tested materials.

**Table 2:** Description of the different experiments carried out. The two right-hand columns represent the number of slides per reactor that has been processed for morphological or microbiological characterization, respectively.

Aim	Experiment Name	Number of CTR	Wall shear stress (Pa)	Slides per CTR for morphological investigations	Slides per CTR for microbiological investigations
Variability	Var 1	1	7.3	4 PP + 4 PVC	6 PP + 6 PVC
	Var 2	1	0.09	4 PP + 4 PVC	6 PP + 6 PVC
Impact of shear stress	Shear 1	3	0.09–3.7–7.3	2 PP	4 PP
	Shear 2	3	0.09–0.79–7.3	3 PP	3 PP
Material	Mat	2	0.09–7.3	2 PP + 2 PVC	3 PP + 3 PVC

The different experimental designs are summed up in Table 2, in which the number of reactors simultaneously operated, the corresponding support material and the wall shear stresses tested are described. The phase and objective of each experiment is reported as well.

The first phase (phase 1) consisted in checking the variability of the biofilms grown simultaneously in one reactor. By demonstrating that two slides placed in the same reactor had a satisfying repeatability, it was possible to reduce the required number of slides per operating condition (*i*.*e*., per reactor) and to increase the number of reactors for phases 2 and 3. The total number of analyzed slides was the same from one experiment to another, but their repartition changed depending on the number of CTR in operation.

In phase 1, two runs (Var 1 and Var 2) were carried out in order to investigate the variability in the adhesion on the two substrata (PP and PVC) at the extreme wall shear stresses of 0.09 and 7.3 Pa. Then, four wall shear stresses ranging from 0.09 to 7.3 Pa were tested to investigate the impact of hydrodynamic strengths on adhesion for PP substratum (phase 2). A first run was carried out with three wall shear stresses: 0.09, 7.3 and an intermediate value of 3.7 Pa. In a second run, 0.09 and 7.3 Pa were repeated to be used as references and the intermediate value was changed to 0.79 Pa. Finally, in phase 3 another material, PVC, was used to confirm the results obtained on PP. Two CTR, operated at different wall shear stresses– 0.09 and 7.3 Pa–and containing both material slides, were carried out, and PP slides were used as control. The effect of both shear stress and material on the adhesion was investigated.

## 3. Results

The variability tests performed on both materials at 7.3 and 0.09 Pa (phase 1) are presented as Supporting Information (S1 Fig and S1 Table). They show that slides of the same material placed in the same reactor were not significantly different for both morphological and microbiological parameters.

### 3.1 Impact of the wall shear stress on the microbial adhesion

During this second phase, only PP material was used as a substratum as PP slides demonstrated more repeatable results. Two runs were carried out, Shear 1 and Shear 2, allowing to test four different wall shear stresses ranging from 0.09 to 7.3 Pa (Table 2). For each experiment, three CTR were simultaneously run, each of them containing six slides. For Shear 1 experiment, two slides per CTR were dedicated to morphological characterization and four slides for microbiological treatment. For Shear 2 experiment, three slides respectively were used for both kinds of characterization (Table 2).

#### 3.1.1. Morphological structure

**Fig 1:**
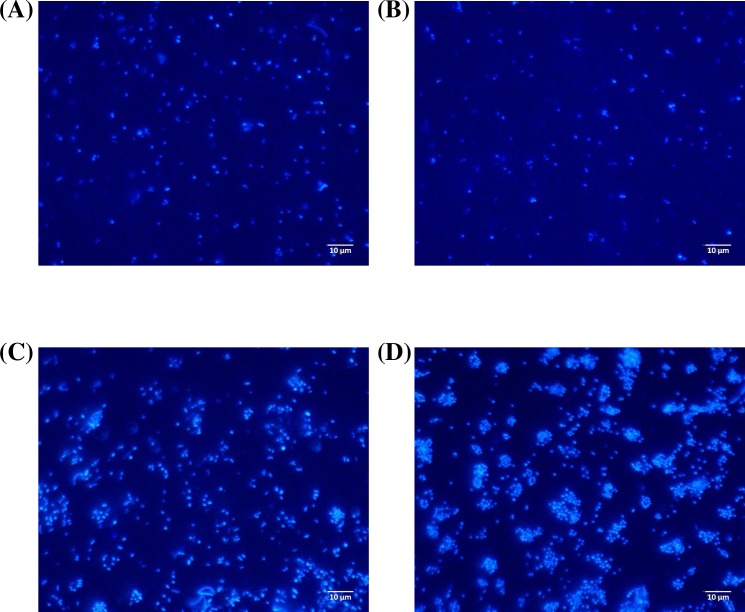
Epifluorescence microscopic pictures acquired during Phase 2, Shear 1 and Shear 2 experiments. Different wall shear stresses were tested: 0.09 Pa (A), 0.79 Pa (B), 3.7 Pa (C) and 7.3 Pa (D). Pictures were obtained with a 60x water immersion objective.

**Fig 2:**
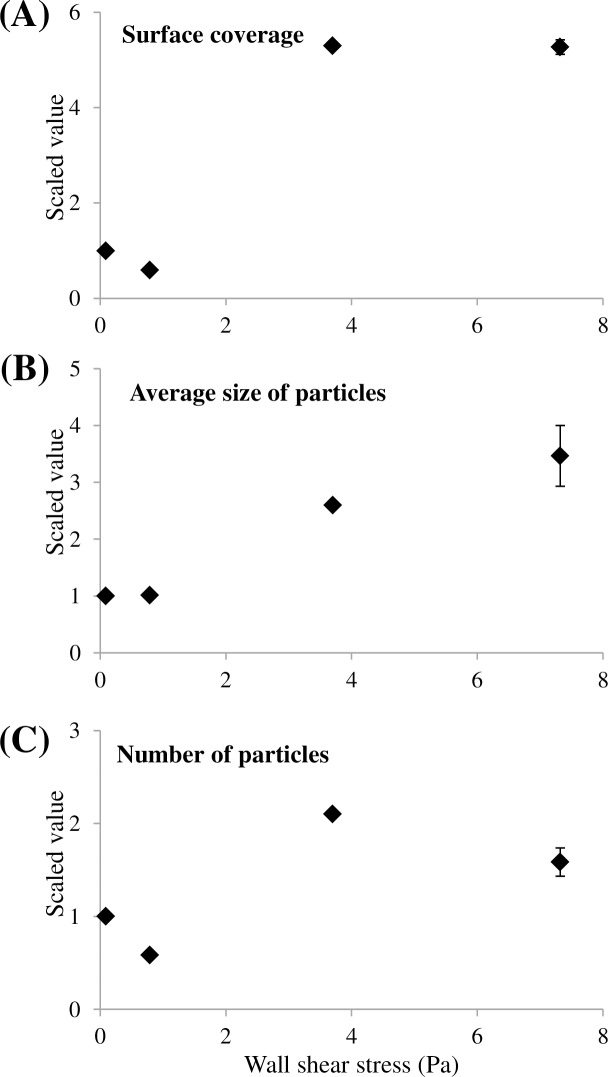
Results of the morphological characterization for Phase 2, shear stress impact test. Three morphological parameters–surface coverage (A), number of particles (B), average size of particles (C)–are reported for the different wall shear stresses tested (Shear 1 and Shear 2 experiments). Data result from microscopic observations and image analysis of acquired pictures. As mentioned in Table 2, two and three slides of PP were characterized per reactor for Shear 1 and Shear 2, respectively. In order to compare on the same graph the four wall shear stresses tested, original values were scaled by the value obtained at 0.09 Pa shear. The y-axis represents this ratio, hence it is dimensionless.

In this section, data were collected on two and three independent replicas (slides) per CTR in Shear 1 and Shear 2 experiments, respectively (Table 2). Fig 1 shows four representative microscopic pictures observed at the four shears. In addition, Fig 2 presents the results for the three parameters describing the physical structure of the microbial adhesion. For each graph, four points corresponding to the four wall shear stresses– 0.09, 0.79, 3.7, 7.3 Pa–are presented. Since the adhesion characteristics were obtained thanks to two successive runs (Shear 1 and Shear 2 experiments), all the values were scaled by using the results obtained at 0.09 Pa as reference. As a consequence, for a given parameter, values obtained for 3.7 Pa and 0.79 Pa were divided by the ones obtained at 0.09 Pa in Shear 1 and Shear 2 experiments, respectively. Therefore, for 7.3 Pa, two experimental results were measured (one per experiment) and the average values with the standard deviations are represented. This procedure aimed at graphically represent all four shear stresses on the same panel despite the fact data originated from different runs. However, statistical tests (ANOVA and t-tests whose p-values are mentioned above) were performed on unscaled original values coming either from one experiment or the other. Original means obtained from unscaled results are available in the Supporting Information S2 Table.

ANOVA tests were performed, demonstrating a highly significant impact of the shear stress on both quantitative (*i*.*e*., the surface coverage) and qualitative aspects (*i*.*e*., number and size of particles) with p-values lower than 10^−16^, for both Shear 1 and Shear 2 experiments respectively.

The increase in the surface coverage with increasing the shear is visualized in Fig 1. Pictures 1a and 1b, corresponding to the lowest shear stresses, present a surface coverage much lower than pictures at 3.7 and 7.3 Pa. This is confirmed with Fig 2A. The two lowest shear stresses had low and similar values of surface coverage, but they were five times higher at 3.7 and 7.3 Pa. As a consequence, shear stress triggered a non-linear increase in the quantity of attached bacteria until a plateau was reached.

In addition to these quantitative aspects, Fig 1 illustrates a change in the distribution pattern of cells on the substratum surface. Single cells were randomly scattered on the surface at 0.09 Pa and at 0.79 Pa. However, at a shear stress of 3.7 Pa, the picture shows that bacteria gathered and formed small clusters even if isolated attached bacteria remained numerous. Since during the adhesion phase the liquid solution was depleted in nutrients, the formation of clusters was only related to aggregation of cells, according to the shear stress and not to the growth of microorganism. Therefore, these aggregates are considered as clusters, and not as micro-colonies (whose formation is linked to growth). At 7.3 Pa, single bacterial cells were still observed, but their number significantly decreased. Most of the surface coverage consisted of bacteria gathered as clusters.

The change in the distribution pattern and the formation of clusters are also highlighted on Fig 2. The variation of the average size of attached particles was linear, with R^2^ equal to 0.96 (Fig 2B). The size of the detected particles increased with the shear. On the other hand, the variation of the number of detected particles was unexpected (Fig 2C). The maximum value was obtained at 3.7 Pa, with twice as particles as at 0.09 Pa and even 20% higher than at 7.3 Pa. It should be noted that at 3.7 and 7.3 Pa, the surface coverages were not significantly different, with approximately 6.29 and 6.08%, respectively, and an ANOVA p-value of 0.37. However, the number and size of particles differed significantly, with p-values of 5x10^-9^ and 3x10^-7^, respectively. A higher number of particles may indeed compensate a lower average size, leading to similar surface coverages. In fact, well-formed clusters, made of several bacteria, were counted as one particle of greater size. The formation of bigger clusters at 7.3 Pa explains why the adhesion presented a pattern with less detected particles but with an average size higher than at 3.7 Pa.

Thus, morphological analysis demonstrated a clear variation in the spatial distribution when increasing the wall shear stress, from a single-cell pattern to a cluster pattern. The average size of particles increased with the shear, highlighting the intensification of this phenomenon.

#### 3.1.2 Microbiological structure

**Fig 3:**
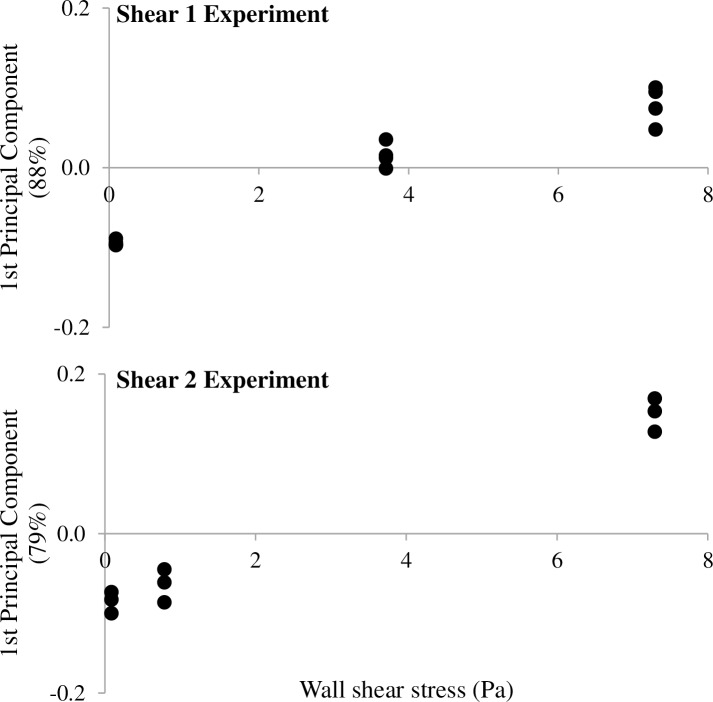
Results of the microbial community characterization for Phase 2, shear stress impact test. Attached bacterial communities on PP slides with wall shear stress tested for Shear 1 (A) and Shear 2 (B) experiments are presented. Each point (●) corresponds to one slide. As mentioned in Table 2, four and three slides of PP were characterized per reactor for Shear 1 and Shear 2, respectively. One slide is missing (Shear 1, 0.09 Pa) because the DNA extraction did not succeed, hence three replicates are represented on the plot. Y-axes are the first Principal Component resulting from the PCA performed on each experiment. For Shear 1 and Shear 2, the first Principal Component sums up 88% and 79% respectively of the total information that are contained in SSCP profiles.

The impact of the shear stress on the bacterial communities was also investigated. Data were collected on four and three independent replicas (slides) per CTR in Shear 1 and Shear 2 experiments, respectively (Table 2). One Principal Component Analysis (PCA) per experiment was performed on SSCP profiles coordinates. Results are presented in Fig 3. On each graph, y-axis corresponds to the first principal component which gathers 88% and 79% of the information contained in the SSCP profiles for Shear 1 and Shear 2 experiments, respectively. Given the low percentage of information aggregated on the second component PC2 (8.5 and 9.7% for Shear 1 and Shear 2 experiments, respectively), only PC1 is used for the representation of the data that in exchange considerably improves the accessibility of the results. Nevertheless, a version of Fig 3. as biplots consisting of PC1 and PC2 is available in the Supporting Information S2 Fig. In addition, original PCR-SSCP profiles of inocula and slides are available in the Supporting Information S3 Fig.

For experiments Shear 1 (Fig 3A) and Shear 2 (Fig 3B), a progressive change of the attached communities with the increasing shear is observed. The distribution of the profiles was found to be strongly dependent on the shear stress applied during the adhesion experiment.

### 3.2 Impacts of wall shear stress and material on the microbial adhesion

In the third phase, in order to check the impact of the wall shear rate on the adhesion on different materials, PP and PVC slides were used (Mat experiment in Table 2). PP and PVC are common plastic materials used in industrial processes and they present distinct adhesion characteristics [4]. As the number of slides per reactor increased when studying these materials, only two reactors were carried out at the extremes wall shear stresses of 0.09 and 7.3 Pa. Each CTR contained ten slides, five of PVC and five of PP. Among these five slides, two were dedicated to morphological characterization and three were used for the microbiological characterization (Table 2).

#### 3.2.1. Morphological structure

**Fig 4:**
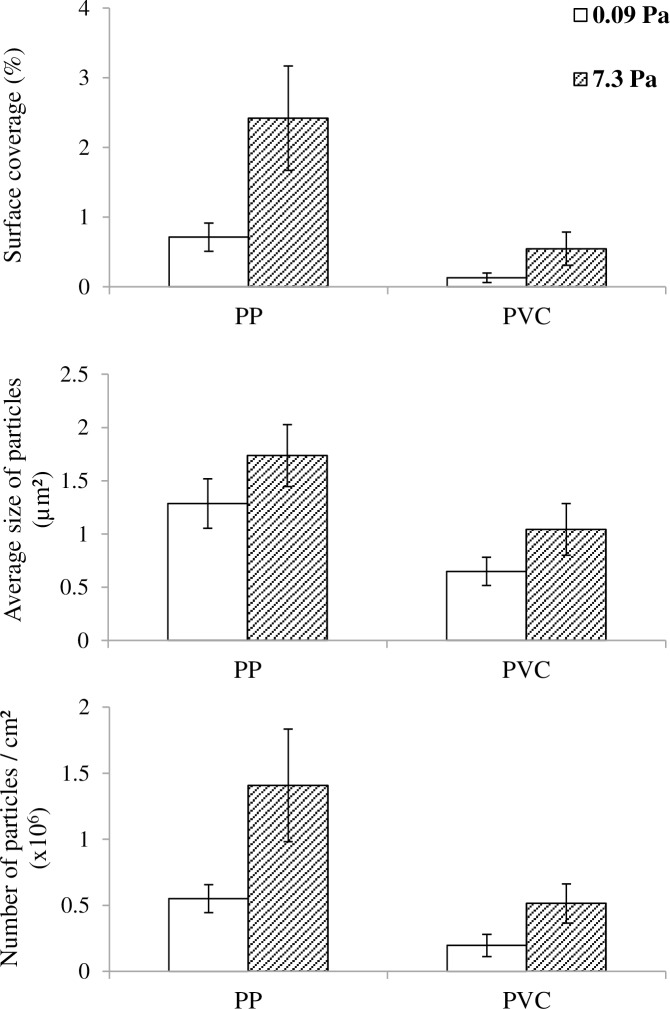
Results of the morphological characterization for Phase 3, material test. Bar plots representing the surface coverage (A), the average size of particles in μm^2^ (B) and the number of particles per cm^2^ (C) obtained for both materials PP and PVC (Mat experiment). Empty bars are for low wall shear stress (0.09 Pa) and hatched bars are for high wall shear stress (7.3 Pa). As mentioned in Table 2, two slides of PP and two slides of PVC were characterized per reactor. Standard variations are represented. They were calculated from the gathering of the 120 pictures recorded from the two slides.

The surface coverage, the number and the average size of detected particles were characterized for PP and PVC slides at both wall shear stresses (Fig 4). For these results, data were collected on two independent replicas (slides) per CTR and per material (Table 2). For PP, surface coverage at high shear was 3.5 times higher than at low shear, confirming the previous results. This difference between surface coverages at high and low shear stress was more pronounced for PVC with a 4.2-fold increase. This increase was explained for both materials by an increase in the average size and number of particles. For PP, the average size increased by 35% while the number of particles had a 150% increase from low to high shear. Similar trends were observed for PVC, with increases of 60% and 160% for size and number of particles, respectively. Welch’s t-tests were performed on each parameter to compare low and high shear values and a high level of significance was obtained for all tests (p-value lower than 10^−16^). As a consequence, the impact of the shear in both qualitative and quantitative microbial adhesion features was confirmed on PVC material, with an increase in the quantity of attached bacteria (i.e. surface coverage) and in the size of detected particles, highlighting the formation of clusters.

The comparison of the adhesion characteristics on both materials at a given shear stress is also interesting. Based on previous work performed by our laboratory [4], PP was considered as a slightly rougher material and with a significantly lower surface energy than PVC, as shown by both non polar and polar components γ^LW^ and γ^AB^, respectively (Table 1). Regarding the microbial adhesion, PP substratum presented higher values for all three parameters at both shears. Welch’s t-tests were also performed and demonstrated a high significance of the impact of the material on the adhesion. These data confirm the results obtained in a previous work [4], where materials with lower surface energy were associated to higher surface coverages. Var 1 and Var 2 also presented similar trends (data not shown). Altogether, these data demonstrate that: (i) the impact of the shear observed on PP was reproducible to another material (PVC) with different surface energy properties and (ii) both the material and the shear had a significant effect on the quantitative and qualitative features of the early biofilm morphology.

#### 3.2.2. Microbiological structure

**Fig 5:**
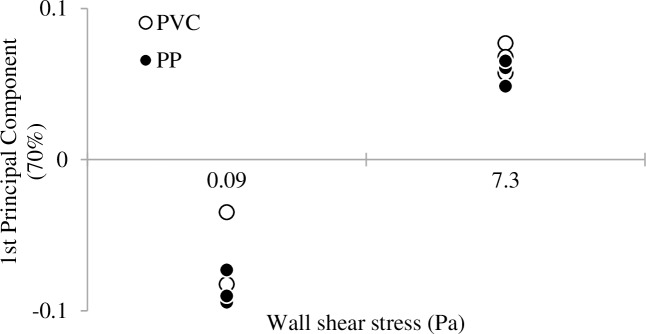
Results of the microbial community characterization for Phase 3, material test. Principal Component plot performed on the bacterial communities harvested on slides of both PP and PVC obtained for material test (Mat experiment). As mentioned in Table 2, three slides of PP and three slides of PVC were characterized per reactor. One slide is missing (PVC, 0.09 Pa) because the DNA extraction did not succeed, hence two replicates are represented on the plot. Euclidian distances between points are proportional to genetic differences between communities.

Fingerprinting of the attached bacterial communities was also determined for each material and shear. Data were collected on three independent replicas (slides) per CTR and per material (Table 2). As for Fig 3 and Fig 5 represents the Principal Component plot performed on SSCP profiles, where only the first Principal Component (70%) is represented (PC2 equaled 15%). Nevertheless, a biplot consisting of PC1 and PC2 is available in the Supporting Information S2 Fig. In addition, original PCR-SSCP profiles of inoculum and slides are available in the Supporting Information S3 Fig. The communities adhered at the low or high shear stresses are well separated along the first Principal Component, indicating a strong impact of the shear on the structure of the microbial community adhered on both PVC and PP materials. However, the nature of the plastic materials tested did not have a significant impact on the adhesion of microorganisms on its surface.

## 4. Discussion

Many studies reported the impact of wall shear stress on bacterial adhesion [9,12,13,32]. However, most of them were realized under laminar regime and with pure cultures, conditions that are not representative of industrial and environmental processes [16,33,34]. Thus, this study focused on the adhesion step under turbulent flow (Reynolds number ranging from 5,000 to 72,000) and involving a complex consortium of microorganisms. The objective was to investigate the influence of the wall shear stress on the microbial adhesion, the spatial distribution of bacteria on the substratum and the structure of the attached microbial communities.

As reported in the literature [1], biofilm formation starts with microbial adhesion, which can be divided in two steps. The first step consists of physical movements that allow the bacteria to reach the substratum surface. Many forces are involved in this step, such as diffusion, gravity, motility or hydrodynamic forces. Then, the microorganisms create bonds on the substratum surface during a second step. The extended Derjaguin-Landau-Verwey-Overbeek (XDLVO) models [23], initially thought for colloidal particles, are widely used to describe and understand this step. Forces such as Van der Waals forces, hydrogen bonds, electrostatic interactions and many others play a key role.

The present work demonstrates that the adhesion is quantitatively enhanced by high wall shear stresses. The improvement of the mass transport promoted the adhesion, facilitating the access of bacteria to the surface of the substratum, the first step of bacterial adhesion. However, a plateau was reached at 3.7 and 7.3 Pa, values showing similar surface coverages. This could be explained by the increase of the detachment forces due to high wall shear rates, jeopardizing the creation of sustainable bonds, the second step of bacterial adhesion. These detachment forces can compensate the positive effect of the mass transport. The ambivalent effect of more intense hydrodynamic conditions on adhesion is well known [11]. It increases the access to the substratum thanks to a more efficient mass transport, but also promotes detachment by increasing the shear. The two opposite mechanisms might explain the similar values obtained at 3.7 and 7.3 Pa, both effects compensating each other.

Nevertheless, despite this quantitative resemblance, differences in the spatial distribution were found. Clusters were identified for higher shears and their size increased when increasing the shear stress. Similar behaviors have already been described [9,35]. Co-adhesion (bacteria attached preferentially to other bacteria already fixed on the substratum forming a growing cluster) and/or co-aggregation (aggregates already formed in the liquid phase and then attached to the substratum) phenomena might have been involved in this clusterisation process [5,36]. However, this kind of microbial interactions, especially in a diverse bacterial consortium as in this work, is still poorly studied and understood [37].

In addition to these morphological considerations, the obtained data demonstrated that different microbiological community structures can occur depending on the shear applied. The change in the bacterial communities is gradual according to the shear magnitude. This could be explained by the different constraints that bacteria have to overcome to be irreversibly attached to the substratum surface. As mentioned previously, bacteria have to reach the substratum surface, and then create chemical bonds with the substratum to make the adhesion sustainable. The ability of bacteria to (i) reach the substratum and (ii) overcome the energy barrier to develop an irreversible adsorption depends on many parameters and the shear is one of them. This could explain why we observed different communities on the slides from the same inoculum (data not shown) and a gradual change in the bacterial communities with the shear. A gradual change in the parameters driving the bacterial selection of the adhesion process is very likely to be acting as the wall shear stress develops. As the mass transport increases, the access to the substratum is facilitated when compared to a low mixing situation. So, more bacteria can reach the substratum, but in return they have to create strong bonds to overcome the associated shear stress. As a consequence, a change in the attached community can be identified, following a change in the selective advantages. Specific assets such as shear dependent adhesion [12], cell shape [38] or affinity for the substratum [7] might represent critical factors explaining the changes in the bacterial community with the applied shear.

The present data also exhibit quantitative differences in adhesion between PP and PVC materials, probably because of different affinities of the microorganisms for these materials. The quantity of bacteria attached on PP substratum was much higher. This corroborates data found in the literature [2,4,7], indicating that rougher and more hydrophobic materials are more suitable for microbial adhesion. In the presence of an anaerobic consortium, it was also demonstrated that PP and PVC materials presented significant differences in the structures of the adhered microbial communities [4]. PP also presented a higher number of adhered microorganisms than PVC. It can be noted here that both materials can be considered as smooth when compared to industrial materials and differences in adhesion between PP and PVC were explained by differences in the surface energy. Nevertheless, in the present work, despite these quantitative differences in terms of adhered microorganisms, similarities concerning the impact of shear stress on qualitative characteristics of adhesion (*i*.*e*., size and distribution of particles) were observed between both PP and PVC materials. The different surface characteristics of both materials did not seem to affect the clusterisation process at high shear stresses. The preponderance of the shear impact over the material impact is also highlighted in terms of attached microbial community, as shown by Fig 5.

In summary, the present work suggests that the wall shear rate can strongly impact the biofilm formation step in terms of microbial community selection and adhesion kinetics and pattern. The presented experimental results were carried out with a diverse inoculum and therefore offer the possibility of assessing the microbial interactions and selection due to shear in the adhesion process. These data suggest that wall shear stress can be a selective lever to promote or prevent the adhesion of a bacterial community. This could be very useful when biofilm is used as a tool such as in environmental biotechnology, where specific populations are expected to grow. In addition, these results show both quantitative and qualitative impacts of the shear on the morphological aspects of adhesion. It would be very interesting to assess the respective contribution of co-adhesion, co-aggregation or bacterial motility on the surface of the substratum known as twitching in the elaboration of the adhesion pattern. Besides being preliminary, these are promising results to either prevent the development of detrimental biofilms or to optimize biofilm-based processes. Further work should however be done to determine whether these differences are smoothed or maintained during biofilm maturation.

## Conclusions

- The number of attached bacteria globally increased with the wall shear stress following a nonlinear relationship. Surface coverages obtained at high shear stresses, 3.7 and 7.3 Pa, exhibited a 5-fold increase compared to values observed at low wall shear stresses, i.e. 0.09 and 0.79 Pa.
- Different spatial distributions were also observed. A single cell adhesion pattern was identified for low wall shear stresses. But as the shear increased, a cluster adhesion pattern appeared. Clusters formed and their size increased with the shear.
- Wall shear stress had a strong impact on the attached bacterial communities. A gradual change in the attached microbiological community with the applied wall shear stress has been identified.
- Adhesion is favored on PP, exhibiting a lower surface energy, rather than on PVC. However, despite the different properties of the materials, similar morphological and microbiological structures were obtained on PVC for the 0.09 and 7.3 Pa wall shear stresses.

## Supporting information

S1 Table Results of variability experiments (phase 1) for morphological data. Coefficients of variation based on four slides of each material (PP and PVC) per condition (i.e. wall shear stress). For each slide, 60 images were recorded which was enough to create a 95% confidence interval with a margin of error of 10% of the mean. Values were slightly higher for PVC material, with one maximum at 26.3%. This is due to the heterogeneity that can exist between two slides of a same manufacturing run.
(PDF)

S2 Table Original data from morphological structure characterization for Shear 1, Shear 2 and Mat experiments. The two following tables give the original non-scaled results (mean and standard variation) for Shear 1 and Shear 2 experiments. The third table presents as a table data illustrated on Fig 4.
(PDF)

S1 Fig Results of variability experiments (phase 1) for microbiological data. SSCP profiles corresponding to the different tested conditions: PP material (A and C) and PVC material (B and D) at 0.09 Pa (A and B) and 7.3 Pa (C and D). Only four PVC profiles were obtained at 7.3 Pa because of unsuccessful DNA extractions on slides PVC3 and PVC4. The repeatability for the bacterial communities was also investigated. Six slides of each material placed under the same shear stress were analyzed with the CE-SSCP fingerprinting technique. For each graph, SSCP profiles overlaid very well, highlighting a good repeatability for both materials and shear.
(PDF)

S2 Fig Biplots of the PCA performed on Shear 1, Shear 2 and Mat experiments, respectively. The three following figures present the microbiological characterization of the attached community on the slides for Shear 1, Shear 2 and Mat experiments. Results are presented as biplots with the x-axis being the first principal component and the y-axis being the second principal component. All replicas are shown.
(PDF)

S3 Fig Original PCR-SSCP profiles of Shear 1, Shear 2 and Mat experiments. The three following figures present the microbiological characterization of inoculum and attached bacteria on the slides for Shear 1, Shear 2 and Mat experiments. All replicas are shown.
(PDF)

## References

[1] Liu Y, Tay J-H. The essential role of hydrodynamic shear force in the formation of biofilm and granular sludge. Water Res. 2002;36: 1653–1665. 12044065 10.1016/s0043-1354(01)00379-7

[2] Habimana O, Semião AJC, Casey E. The role of cell-surface interactions in bacterial initial adhesion and consequent biofilm formation on nanofiltration/reverse osmosis membranes. J Memb Sci. Elsevier; 2014;454: 82–96.

[3] Nicolella C, van Loosdrecht MCM, Heijnen JJ. Wastewater treatment with particulate biofilm reactors. J Biotechnol. 2000;80: 1–33. 10862983 10.1016/s0168-1656(00)00229-7

[4] Habouzit F, Gévaudan G, Hamelin J, Steyer J-P, Bernet N. Influence of support material properties on the potential selection of Archaea during initial adhesion of a methanogenic consortium. Bioresour Technol. 2011;102: 4054–60. 10.1016/j.biortech.2010.12.023 21211965

[5] Bos R, van der Mei HC, Meinders JM, Busscher HJ. A quantitative method to study co-adhesion of microorganisms in a parallel plate flow chamber: basic principles of the analysis. J Microbiol Methods. 1994;20: 289–305.

[6] Andrews CS, Denyer SP, Hall B, Hanlon GW, Lloyd a W. A comparison of the use of an ATP-based bioluminescent assay and image analysis for the assessment of bacterial adhesion to standard HEMA and biomimetic soft contact lenses. Biomaterials. 2001;22: 3225–33. 11700794 10.1016/s0142-9612(01)00160-0

[7] Meylheuc T, Methivier C, Renault M, Herry J-M, Pradier C-M, Bellon-Fontaine MN. Adsorption on stainless steel surfaces of biosurfactants produced by gram-negative and gram-positive bacteria: consequence on the bioadhesive behavior of Listeria monocytogenes. Colloids Surf B Biointerfaces. 2006;52: 128–37. 10.1016/j.colsurfb.2006.04.016 16781848

[8] Florjanič M, Kristl J. The control of biofilm formation by hydrodynamics of purified water in industrial distribution system. Int J Pharm. 2011;405: 16–22. 10.1016/j.ijpharm.2010.11.038 21129467

[9] Perni S, Jordan S, Andrew P, Shama G. Biofilm development by Listeria innocua in turbulent flow regimes. Food Control. 2006;17: 875–883.

[10] Brugnoni L, Cubitto M, Lozano J. Candida krusei development on turbulent flow regimes: Biofilm formation and efficiency of cleaning and disinfection program. J Food Eng. 2012;111: 546–552.

[11] Busscher HJ, van der Mei HC. Microbial adhesion in flow displacement systems. Clin Microbiol Rev. 2006;19: 127–141. 10.1128/CMR.19.1.127-141.2006 16418527 PMC1360269

[12] Lecuyer S, Rusconi R, Shen Y, Forsyth A, Vlamakis H, Kolter R, et al Shear stress increases the residence time of adhesion of Pseudomonas aeruginosa. Biophys J. Biophysical Society; 2011;100: 341–350. 10.1016/j.bpj.2010.11.078 PMC3021681 21244830

[13] Park A, Jeong H-H, Lee J, Kim KP, Lee C-S. Effect of shear stress on the formation of bacterial biofilm in a microfluidic channel. BioChip J. 2011;5: 236–241.

[14] Rochex A, Godon J-J, Bernet N, Escudié R. Role of shear stress on composition, diversity and dynamics of biofilm bacterial communities. Water Res. 2008;42: 4915–4922. 10.1016/j.watres.2008.09.015 18945468

[15] Bakker D, Van der Plaats A, Verkerke G, Busscher H, Van der Mei H. Comparison of velocity profiles for different flow chamber designs used in studies of microbial adhesion to surfaces. Appl Environ Microbiol. 2003;69: 6280–6287. 10.1128/AEM.69.10.6280-6287.2003 14532092 PMC201240

[16] Wang H, Sodagari M, Chen Y, He X, Newby B-MZ, Ju L-K. Initial bacterial attachment in slow flowing systems: Effects of cell and substrate surface properties. Colloids Surfaces B Biointerfaces. 2011;87: 415–22. 10.1016/j.colsurfb.2011.05.053 21715146

[17] Lelièvre C, Legentilhomme P, Gaucher C, Legrand J, Faille C, Bénézech T. Cleaning in place: effect of local wall shear stress variation on bacterial removal from stainless steel equipment. Chem Eng Sci. 2002;57: 1287–1297.

[18] Ochoa J-C, Coufort C, Escudié R, Liné A, Paul E, Escudie R, et al Influence of non-uniform distribution of shear stress on aerobic biofilms. Chem Eng Sci. 2007;62: 3672–3684.

[19] Characklis WG, Marshall KC, (eds). Biofilms. New-York, USA: John Wiley and Sons; 1990.

[20] Andereck CD, Liu SS, Swinney HL. Flow regimes in a circular Couette system with independently rotating cylinders. J Fluid Mech. 1986;164: 155–183.

[21] Kataoka K. Chapter 9—Taylor vortices and instabilities in circular Couette flows Encyclopedia of fluid mechanics, Volume 1: Flow phenomena and measurements. Houston: Gulf Publishing Co; 1986 pp. 236–274.

[22] Racina A, Kind M. Specific power input and local micromixing times in turbulent Taylor–Couette flow. Exp Fluids. 2006;41: 513–522.

[23] Van Oss CJ. Hydrophobicity of biosurfaces—Origin, quantitative determination and interaction energies. Colloids Surfaces B Biointerfaces. 1995;5: 91–110.

[24] Taylor GI. Stability of a Viscous Liquid Contained between Two Rotating Cylinders. Philos Trans R Soc A Math Phys Eng Sci. 1923;223: 289–343.

[25] Fenstermacher P, Swinney HL, Gollub J. Dynamical instabilities and the transition to chaotic Taylor vortex flow. J Fluid Mech. 1979;94: 103–128.

[26] Rasband W. ImageJ, US National Institutes of Health, Bethesda, Maryland, USA 1997;

[27] Wéry N, Bru-Adan V, Minervini C, Delgénes J-P, Garrelly L, Godon J-J. Dynamics of Legionella spp. and bacterial populations during the proliferation of L. pneumophila in a cooling tower facility. Appl Environ Microbiol. 2008;74: 3030–7. 10.1128/AEM.02760-07 18390683 PMC2394956

[28] Milferstedt K, Santa-Catalina G, Godon J-J, Escudié R, Bernet N. Disturbance frequency determines morphology and community development in multi-species biofilm at the landscape scale. PLoS One. 2013;8: e80692 10.1371/journal.pone.0080692 24303024 PMC3841191

[29] Michelland RJ, Dejean S, Combes S, Fortun-Lamothe L, Cauquil L. StatFingerprints: a friendly graphical interface program for processing and analysis of microbial fingerprint profiles. Mol Ecol Resour. 2009;9: 1359–63. 10.1111/j.1755-0998.2009.02609.x 21564907

[30] R Development Core Team. R: a language and environment for statistical computing. 2010.

[31] Oksanen J, Blanchet GF, Kindt R, Legendre P, Minchin PR, O’Hara RB, et al. Vegan: Community Ecology Package [Internet]. 2011. Available: http://cran.r-project.org/package=vegan

[32] Thomas WE, Trintchina E, Forero M, Vogel V, Sokurenko E V. Bacterial adhesion to target cells enhanced by shear force. Cell. Cell Press; 2002;109: 913–923. 12110187 10.1016/s0092-8674(02)00796-1

[33] Moreira JMR, Araújo JDP, Miranda JM, Simões M, Melo LF, Mergulhão FJ. Colloids and Surfaces B: Biointerfaces The effects of surface properties on Escherichia coli adhesion are modulated by shear stress. Colloids Surfaces B Biointerfaces. Elsevier B.V.; 2014;123: 1–7. 25218513 10.1016/j.colsurfb.2014.08.016

[34] Teodósio JS, Simões M, Melo LF, Mergulhão FJ. Flow cell hydrodynamics and their effects on E. coli biofilm formation under different nutrient conditions and turbulent flow. Biofouling. 2011;27: 1–11. 10.1080/08927014.2010.535206 21082456

[35] Brugnoni LI, Cubitto MA, Lozano JE. Role of shear stress on biofilm formation of Candida krusei in a rotating disk system. J Food Eng. 2011;102: 266–271.

[36] Kolenbrander PE, Palmer RJ, Periasamy S, Jakubovics NS. Oral multispecies biofilm development and the key role of cell-cell distance. Nat Rev Microbiol. 2010;8: 471–80. 10.1038/nrmicro2381 20514044

[37] Bos R, van der Mei HC, Busscher HJ. A quantitative method to study co-adhesion of microorganisms in a parallel plate flow chamber. II: Analysis of the kinetics of co-adhesion. J Microbiol Methods. 1995;23: 169–182.

[38] Young KD. The selective value of bacterial shape. Microbiol Mol Biol Rev. 2006;70: 660–703. 10.1128/MMBR.00001-06 16959965 PMC1594593

