# Supplementary material for: Impact of wall shear stress on initial bacterial adhesion in rotating annular reactor
Source: PLoS One. 2017 Feb 16;12(2):e0172113. doi: 10.1371/journal.pone.0172113 (PMC5312967; doi:10.1371/journal.pone.0172113)
Supplement: S1 Table — Coefficients of variation based on four slides of each material (PP and PVC) per condition (i.e. wall shear stress). For each slide, 60 images were recorded which was enough to create a 95% confidence interval with a margin of error of 10% of the mean. Values were slightly higher for PVC material, with one maximum at 26.3%. This is due to the heterogeneity that can exist between two slides of a same manufacturing run. (PDF) [file pone.0172113.s001.pdf]

S1 Table: Results of variability experiments (phase 1) for morphological data. Coefficients of variation based on four slides of each material (PP and PVC) per condition (i.e. wall shear stress).

	Surface coverage		Number of particles		Size of particles	
	PP	PVC	PP	PVC	PP	PVC
VAR1<br>0.09 Pa	9.8 %	26.3 %	5.9 %	16.1 %	12.0 %	17.0 %
VAR2<br>7.3 Pa	8.2 %	10.2 %	10.6 %	11.7 %	7.6 %	13.0 %

For each slide, 60 images were recorded which was enough to create a 95% confidence interval with a margin of error of 10% of the mean. Values were slightly higher for PVC material, with one maximum at 26.3%. This is due to the heterogeneity that can exist between two slides of a same manufacturing run.
