# Supplementary material for: Impact of wall shear stress on initial bacterial adhesion in rotating annular reactor
Source: PLoS One. 2017 Feb 16;12(2):e0172113. doi: 10.1371/journal.pone.0172113 (PMC5312967; doi:10.1371/journal.pone.0172113)
Supplement: S2 Table — The two following tables give the original non-scaled results (mean and standard variation) for Shear 1 and Shear 2 experiments. The third table presents as a table data illustrated on Fig 4. (PDF) [file pone.0172113.s002.pdf]

S2 Table: Original data from morphological structure characterization for Shear 1, Shear 2 and Mat experiments. The two following tables give the original non-scaled results (mean and standard variation) for Shear 1 and Shear 2 experiments. The third table presents as a table data illustrated on Fig.4.

Shear 1	Mean			Standard variation		
	Number of particles / cm²	Size of a particle (µm²)	Surface coverage %	Number of particles / cm²	Size of a particle (µm²)	Surface coverage %
0.09 Pa	8.0E+05	1.44	1.19	3.0E+05	0.29	0.58
3.7 Pa	1.7E+06	3.75	6.29	4.2E+05	0.64	1.81
7.3 Pa	1.4E+06	4.23	6.08	4.2E+05	1.04	2.71

Shear 2	Mean			Standard variation		
	Number of particles / cm²	Size of a particle (µm²)	Surface coverage %	Number of particles / cm²	Size of a particle (µm²)	Surface coverage %
0.09 Pa	1.7E+06	1.48	2.48	3.1E+05	0.24	0.62
0.79 Pa	9.8E+05	1.49	1.48	2.3E+05	0.32	0.49
7.3 Pa	2.4E+06	5.91	13.45	6.7E+05	1.78	3.28

Mat		Mean			Standard variation		
		Number of particles / cm²	Size of a particle (µm²)	Surface coverage %	Number of particles / cm²	Size of a particle (µm²)	Surface coverage %
PP	0.09 Pa	5.5E+05	1.29	0.71	1.1E+05	0.23	0.20
	7.3 Pa	1.4E+06	1.74	2.42	4.3E+05	0.29	0.75
PVC	0.09 Pa	2.0E+05	0.65	0.13	8.5E+04	0.13	0.07
	7.3 Pa	5.1E+05	1.04	0.55	1.5E+05	0.24	0.24
