# Supplementary material for: Impact of wall shear stress on initial bacterial adhesion in rotating annular reactor
Source: PLoS One. 2017 Feb 16;12(2):e0172113. doi: 10.1371/journal.pone.0172113 (PMC5312967; doi:10.1371/journal.pone.0172113)
Supplement: S1 Fig — SSCP profiles corresponding to the different tested conditions: PP material (A and C) and PVC material (B and D) at 0.09 Pa (A and B) and 7.3 Pa (C and D). Only four PVC profiles were obtained at 7.3 Pa because of unsuccessful DNA extractions on slides PVC3 and PVC4. The repeatability for the bacterial communities was also investigated. Six slides of each material placed under the same shear stress were analyzed with the CE-SSCP fingerprinting technique. For each graph, SSCP profiles overlaid very well, highlighting a good repeatability for both materials and shear. (PDF) [file pone.0172113.s003.pdf]

S1 Fig: **Results of variability experiments (phase 1) for microbiological data**. SSCP profiles corresponding to the different tested conditions: PP material (A and C) and PVC material (B and D) at 0.09 Pa (A and B) and 7.3 Pa (C and D). Only four PVC profiles were obtained at 7.3 Pa because of unsuccessful DNA extractions on slides PVC3 and PVC4.

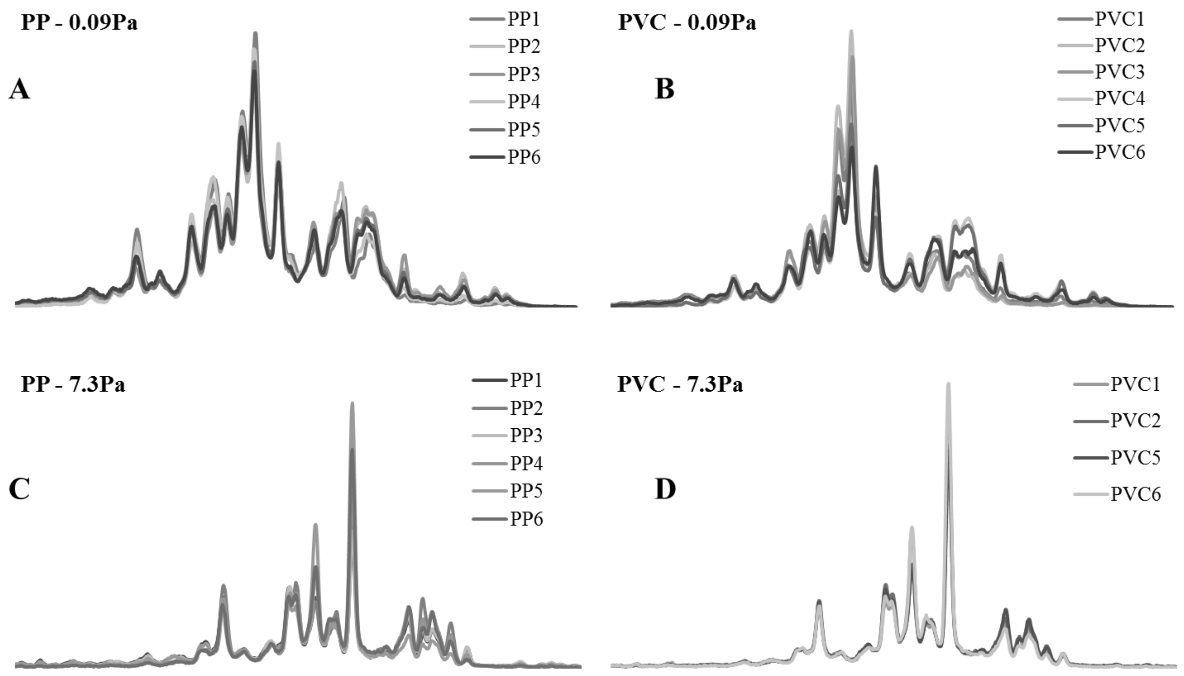

The repeatability for the bacterial communities was also investigated. Six slides of each material placed under the same shear stress were analyzed with the CE-SSCP fingerprinting technique. For each graph, SSCP profiles overlaid very well, highlighting a good repeatability for both materials and shear.
