# Supplementary material for: Impact of wall shear stress on initial bacterial adhesion in rotating annular reactor
Source: PLoS One. 2017 Feb 16;12(2):e0172113. doi: 10.1371/journal.pone.0172113 (PMC5312967; doi:10.1371/journal.pone.0172113)

S2 Figure: Biplots of the PCA performed on Shear 1, Shear 2 and Mat experiments, respectively. The three following figures present the microbiological characterization of the attached community on the slides for Shear 1, Shear 2 and Mat experiments. Results are presented as biplots with the x-axis being the first principal component and the y-axis being the second principal component. All replicas are shown.

Shear 1 experiment:

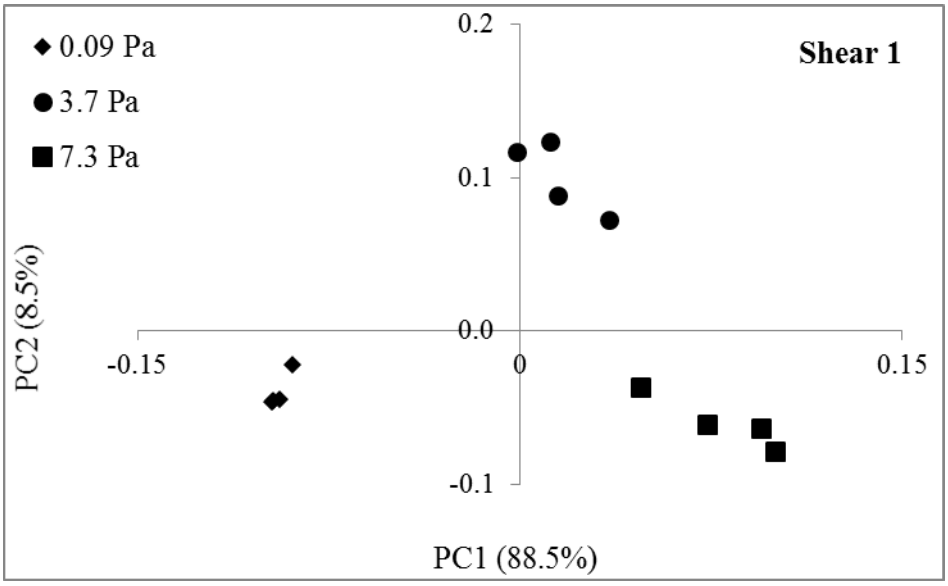

Shear 2 experiment:

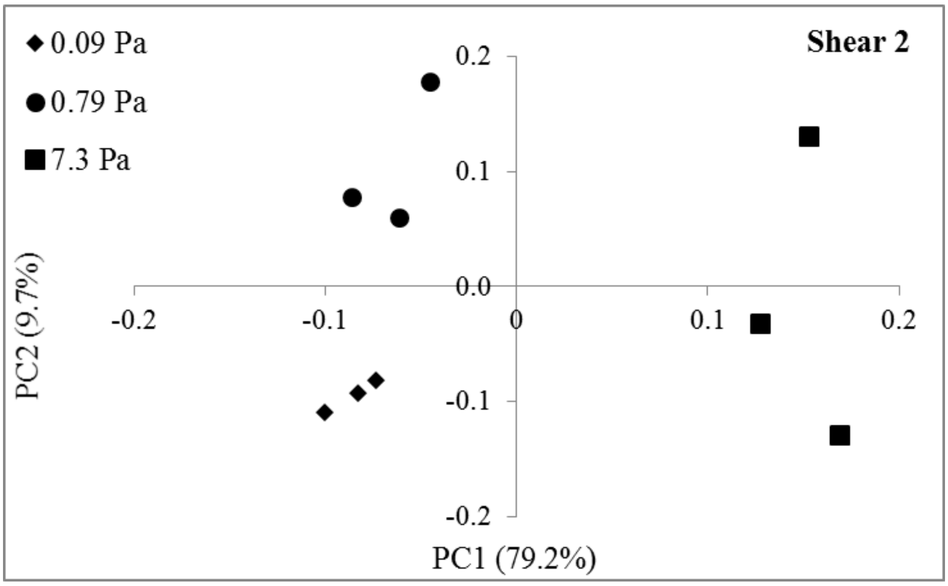

Mat experiment:

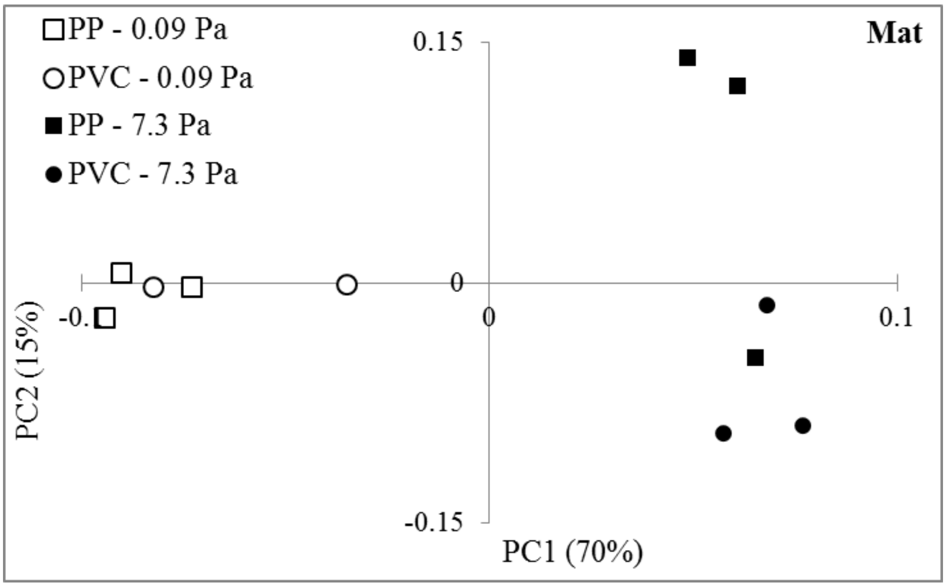

Supplement: S2 Fig — The three following figures present the microbiological characterization of the attached community on the slides for Shear 1, Shear 2 and Mat experiments. Results are presented as biplots with the x-axis being the first principal component and the y-axis being the second principal component. All replicas are shown. (PDF) [file pone.0172113.s004.pdf]
